# ‘From Pilot to production’: Large Scale Digitisation project at Naturalis Biodiversity Center

**DOI:** 10.3897/zookeys.209.3609

**Published:** 2012-07-20

**Authors:** Jon Peter van Oever, Marc Gofferjé

**Affiliations:** 1 NCB Naturalis, 2333 CK, Leiden, Nederlands

**Keywords:** Large scale digitisation, NCB Naturalis, Pilot, Collection, Digistreet, Programme

## Abstract

By the end of 2009 the Dutch Government awarded the establishment of NCB Naturalis with €30M funding. The amount is invested in three programs: Scientific Infrastructure for DNA Barcoding, Integration and Relocation of collections and Collection Digitisation. In this article we describe the highlights of the Digitisation Programme.

## Introduction

Naturalis Biodiversity Center, the Netherlands Center for Biodiversity, was launched on 28 January 2010. The center is the result of the cooperation between Amsterdam University (Amsterdam Zoological Museum), Leiden University and Wageningen University and Research Centre (National Herbarium Netherlands) and the National Natural History Museum Naturalis in Leiden. The partners’ collections are being brought together at Naturalis BC and will be integrated into a collection totalling over 37 million objects. In terms of collection size, Naturalis BC is one of the top five natural history museums in the world.

By the end of 2009 the Dutch Government awarded the establishment of (at that time) NCB Naturalis with €30M funding from the National Gas and oil profits (FES=funding economical structure (empowerment). This fund is responsible for many investments in the Cultural Heritage Sector. The amount is invested in three programs: Scientific Infrastructure for DNA Barcoding, Integration and Relocation of collections and Collection Digitisation. In this article we only describe the highlights of the Digitisation Programme.

## Digitisation program at Naturalis Biodiversity Center

In 2010 the preparations began to develop an overall program for the mass digitisation of the collections. The program organisation had to meet 2 main goals:

– digitise at least 7M objects of the total of 37M specimen/objects;

– develop a permanent digitisation infrastructure (to ensure the remaining objects can be processed in the near future).

The structure by which the digitisation has been developed at Naturalis is different from the classical approach. In the current economic crisis the challenge is to do more with less money. Therefore the solutions must contain new and innovative perspectives on digitisation.

When Naturalis applied for funds, the average cost of digitisation was estimated (by experience of the past) to be approximately € 5 per object. The Dutch government granted € 13 M to digitize approximately 7 million objects (average € 1.86 per object).

Therefore the following decisions had to be made:

• to digitise a large number of objects through an industrial approach.

• To collect only basic metadata associated with an object, which later can be amended.

The Prince 2 methodology is used and the projects timeframe was first set to Q4 – 2013, which was later extended to June 2015. Project governance is carried out by the Steering Committee, overseeing scientific quality of the project. The board of directors of Naturalis BC is represented in the steering committee. Program manager, project managers and project leaders are responsible for everyday work, from the project set up to hiring staff, from housing to planning of collections to operations control, from budgeting to decision preparation and execution. The entire program consists of around 80 people. Several partner institutions (Paris, London, Finland, Berlin) were visited to define best practices. A series of pilot projects were conducted before commencing large-scale digitisation projects and selecting outsourcing partners.

Several stages of the Programme implementation can be distinguished:

• Testing and selecting technologies

• Developing tools: Basic Registration Database and Central Registration System

• Conducting Pilot Projects

• Selection and Prioritization of collections for digitisation

• Choosing outsource vendors and suppliers

• Execution of projects

## Approach

**Figure 1.**
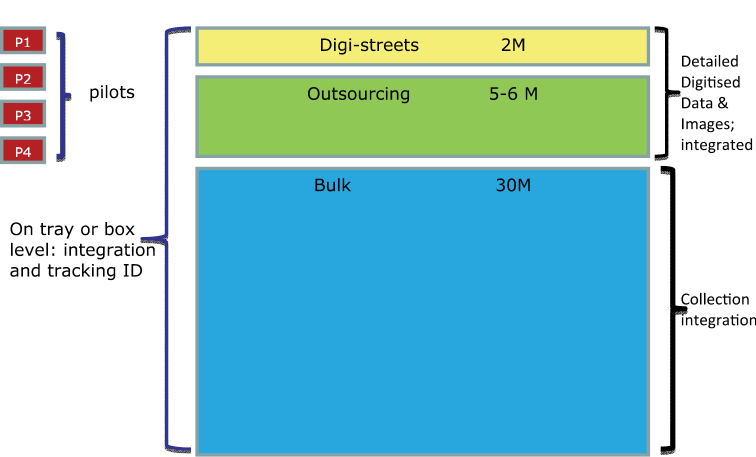
Structure of the digitization programme at Naturalis Biodiversity Center.

A tier-based approach has been developed for digitisation of the Naturalis collection (Fig. 1)

• ~2,000,000 specimens are to be digitised in-house with detailed metadata extracted (“Digistreets”)

• ~5,000,000 specimens will be digitised with basic metadata acquisition through outsourced vendors

• For the rest of the collection (~30,000,000 specimens) a high-level inventory will be created.

## Prioritisation

When selecting parts of the collection for in-house detailed digitisation the most important factor was prioritisation by scientific or outreach value of the outcome (see below). Therefore, collections related to particular research or curatorial activities were identified. Value-for-money was a decisive criterion for outsourcing digitisation. Only collections for which industrial-scale digitisation technologies exist, which can be relatively safely moved to another site, and where such service is provided for reasonable price, were selected. The most obvious example of such collections is herbaria. For collections, which are not extensively used at the present, or for which mass digitisation technologies are not yet available, or too expensive, high-level inventory will be built, describing content of every drawer or lot as detailed as practical.

**Figure 2.**
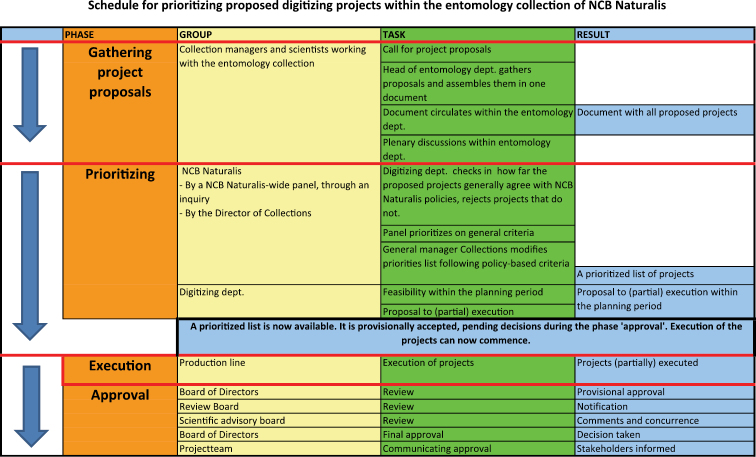
Life cycle of Digistreets: stages of planning and executing of progect.

One of the key strengths of the digistreets is that they must be demand driven and therefore collection independent. The Programme has developed a framework of priority setting and decision making in accordance with the institution’s priorities (Fig. 2). The most treated, most important collections are key for the priority selection. This is a radical change of policy where in the past every scientist, taxonomist or biodiversity researcher had a personal history of raising funds and persuading decision makers into why their project should be prioritised. Transparency of procedure and objectified criteria of selection help to identify priority collections. Some of the indicators are:

• collaborative biodiversity projects

• European-funded and co-funded projects

• economic importance of the group

• relevance for citizen scientists and lay public

• collection conservation status

Prioritisation of projects is a multi-step process and includes (1) prerequisites: criteria mandatory for all projects, to reject unacceptable projects; (2) soft criteria: professional opinion of panel members, to create a long list of candidate projects; and (3) hard criteria: point-base factual criteria, to weigh projects and to arrange them in order of preference.

## Pilots

Every digistreet is developed and derived from a pilot phase. The pilot can be defined as a proof of concept of a particular project or technology. A set of success criteria is devised and agreed upon start of every pilot. A time frame between the 3 to 6 months is needed to sharpen the requirements, the workflow, object handling and to test the technology. The Mollusc and the Entomology digistreet were the first two industrialised production projects developed. The Mollusc digistreet can be visited at the Live Science Hall at the museum. An application for iPads was developed so the visitors can be involved in transcription of scanned label information. Within 9 months 17K labels were transcribed by the members of public and checked by our taxonomists. Approximately 8K label transcriptions were useful. The data will be imported in the system. The App is enhanced and web enabled and now available for the visitors at the NCB website. The idea is that in the near future every digistreet will have an App to engage the community. After the pilot phase an evaluation report is constructed for the steering committee which made a decision on viability of a larger project. Most of the pilots were transferred into digistreets. A few pilots have not been developed to full-scale projects because the technology or process didn’t meet the quality standards or requirements. An example was the 3D digitization of Bird’s specimen. The quality of the images and the 3d viewing technologies were not mature enough.

## Digistreets

‘Digistreets’ are production lines for digitisation of objects that have a lot in common from the point of view of registration, handling, and safety regulations.

Based on the overall collection characteristics nine digistreets were defined and developed:

• Wood samples

• Entomology collections

• Herbarium sheets

• Mollusc collections

• Dry mounted Vertebrates/Invertebrates;

• Alcohol/formaldehyde samples

• Microscopic slides;

• 2-D material (drawings, rare books, photographs, paintings, archives, microfiches etc.);

• Geological and paleontological collections

Each digistreet is managed as a separate project; it has a specific location, set of tools and equipment, and a more or less tailored version of the Central Registration System. Fixed targets (scope, time, quality) and a fixed budget are set for each digistreet; and staff are provided for the duration of the project. Every digistreet staff member is fully aware of what they are supposed to process in what time at what cost and quality. An exception is the herbarium which combines the shipment of all the duplicate sheets at Wageningen and the development of two separate production lines: an outsourcing street in Leiden and a digistreet in Wageningen. The experience of The digistreets guidelines and requirements are being applied for the outsourcing part.

The acceptance of the goals of the digitisation project by the organisation is the key to successful projects. A collection manager is needed to instruct and manage the “streetworkers”. Registrators (data entry), taxonomists and teamleaders are managed by the digistreets’ projectleader. A process owner (institutionalised job role) is the leading decision maker on collections policies and priorities. He or she can oversee the individual collection requirements or the demands from the sections Collection Management, Research or Outreach. The process owner is also ultimately responsible for the safety of the staff/people or the collection objects.

## Results

From the start of the programme (August 2010) until July 2012 approximately 1,000,000 objects have been internally digitised by a temporary staff of 80 people employed in digistreets. The outsourcing project, digital image bank and content management system are in a tendering phase and will be implemented in Q3/Q4 2012. Average costs per digitized object is provided in Table 1.

**Table 1.** 

Cost of digistreets, per object	€ 2.50
Cost of outsourcing, per object	€ 0.90
Cost of infrastructure and equipment, per object	€ 0.30
Overhead (project management etc), per object	€ 0.20
Average cost per digitized object, entire programme	**€ 1.86**

## Conclusions

• Mass digitisation of natural history objects is proven to be possible at reasonable costs;

• Industrial methods and concepts are a help—not a threat—to collection management and large scale object digitisation;

• By digitising the collection it is ensured that the data is available online, comparable and validated independently from location and time;

• Through the digitisation process new relations and associations can be made between taxonomies, object transcriptions, meta data, context and images;

• Data is provided using the taxonomic worldwide standards (GBIF, Darwin-core) and can be accumulated, amended and used nationally and internationally.

